# A hybrid-membrane migration method to isolate high-purity adipose-derived stem cells from fat tissues

**DOI:** 10.1038/srep10217

**Published:** 2015-05-13

**Authors:** Akon Higuchi, Ching-Tang Wang, Qing-Dong Ling, Henry Hsin-chung Lee, S. Suresh Kumar, Yung Chang, Abdullah A. Alarfaj, Murugan A. Munusamy, Shih-Tien Hsu, Gwo-Jang Wu, Akihiko Umezawa

**Affiliations:** 1 Department of Chemical and Materials Engineering, National Central University, Jhong-li, Taoyuan, Taiwan; 2 Department of Botany and Microbiology, King Saud University, Riyadh, Saudi Arabia; 3 Department of Reproduction, National Research Institute for Child Health and Development, Tokyo, Japan; 4 Nano Medical Engineering Laboratory, RIKEN, Wako, Saitama, Japan; 5 Institute of Systems Biology and Bioinformatics, National Central University, Jhong-li, Taoyuan, Taiwan; 6 Cathay Medical Research Institute, Cathay General Hospital, Taipei, Taiwan; 7 Department of Surgery, Cathay General Hospital, Taipei, Taiwan; 8 Department of Medical Microbiology and Parasitology, Universities Putra Malaysia, Slangor, Malaysia; 9 Department of Chemical Engineering, R&D Center for Membrane Technology, Chung Yuan Christian University, Jhong-li, Taoyuan, Taiwan; 10 Department of Internal Medicine, Taiwan Landseed Hospital, Pingjen, Taoyuan, Taiwan; 11 Graduate Institute of Medical Sciences and Department of Obstetrics & Gynecology, Tri-Service General Hospital, National Defense Medical Center, Taipei, Taiwan

## Abstract

Human adipose-derived stem cells (hADSCs) exhibit heterogeneous characteristics, indicating various genotypes and differentiation abilities. The isolated hADSCs can possess different purity levels and divergent properties depending on the purification methods used. We developed a hybrid-membrane migration method that purifies hADSCs from a fat tissue solution with extremely high purity and pluripotency. A primary fat-tissue solution was permeated through the porous membranes with a pore size from 8 to 25 μm, and the membranes were incubated in cell culture medium for 15-18 days. The hADSCs that migrated from the membranes contained an extremely high percentage (e.g., >98%) of cells positive for mesenchymal stem cell markers and showed almost one order of magnitude higher expression of some pluripotency genes (*Oct4*, *Sox2*, *Klf4* and *Nanog*) compared with cells isolated using the conventional culture method.

Human adult stem cells, such as human adipose-derived stem cells (hADSCs), are considered to be a more attractive source of stem cells than human embryonic stem cells (hESCs)^1^ and human induced pluripotent stem cells (hiPSCs)^2^,^3^,^4^,^5^. This is because human adult stem cells do not generate the ethical concerns that accompany hESCs. Furthermore, xeno-free cultures of hESCs and hiPSCs are currently difficult to achieve and/or are extremely costly^3^,^6^. Although hADSCs are promising for use in regenerative medicine, their lower differentiation ability due to the lower pluripotency of hADSCs compared with hESCs and hiPSCs is a critical issue. One cause of these defects originates from the heterogeneity of hADSCs. hADSCs are not a homogeneous cell population but rather contain cells with the ability to differentiate into several specific lineages.

In general, hADSCs are isolated from liposuction-derived adipose tissue, digested by collagenase, centrifuged, and then cultivated in cell culture dishes for at least one passage to purify hADSCs (the “culture method”)^7^,^8^,^9^. hADSCs are defined by the expression of specific cell surface markers of mesenchymal stem cells (MSCs), such as CD29, CD44, CD73, CD90, CD105, and CD166^7^,^9^,^10^,^11^. hADSCs can be isolated from and/or characterized by these surface markers using flow cytometry. However, it is extremely difficult to obtain hADSCs that express all of the above surface markers with greater than 90% purity in general. In most cases, first-passage hADSCs have been approximately 60-80% positive for these MSC markers in previous studies^7^,^9^, indicating that hADSCs are not a homogeneous cell population. The clinical application of hADSCs requires an easy and xeno-free method able to purify hADSCs with high purity and high pluripotency.

Specific cells can be purified using several methods, such as fluorescence-activated cell sorting (FACS)^12^,^13^, magnetic-activated cell sorting (MACS)^14^ and membrane filtration^9^. FACS and MACS use antibodies to purify specific target cells but are unfavorable for clinical applications due to the possibility of contamination with viruses or prions. Among these methods, the purification of stem cells by membrane filtration methods is attractive due to its simplicity and sterility.

We previously reported the purification of ADSCs from mouse and human adipose tissue cell solutions using the membrane filtration method^9^,^15^. However, hADSCs purified using membrane filtration were much less pure (i.e., 20-60%) than hADSCs purified by the conventional culture method in the previous study^9^. Therefore, we have developed a hybrid-membrane migration method that is a combination of the membrane filtration method and the membrane cultivation method that enables us to obtain hADSCs with high purity and pluripotency.

## Results

### Isolation of hADSCs by hybrid-membrane migration method

**Figure 1:**
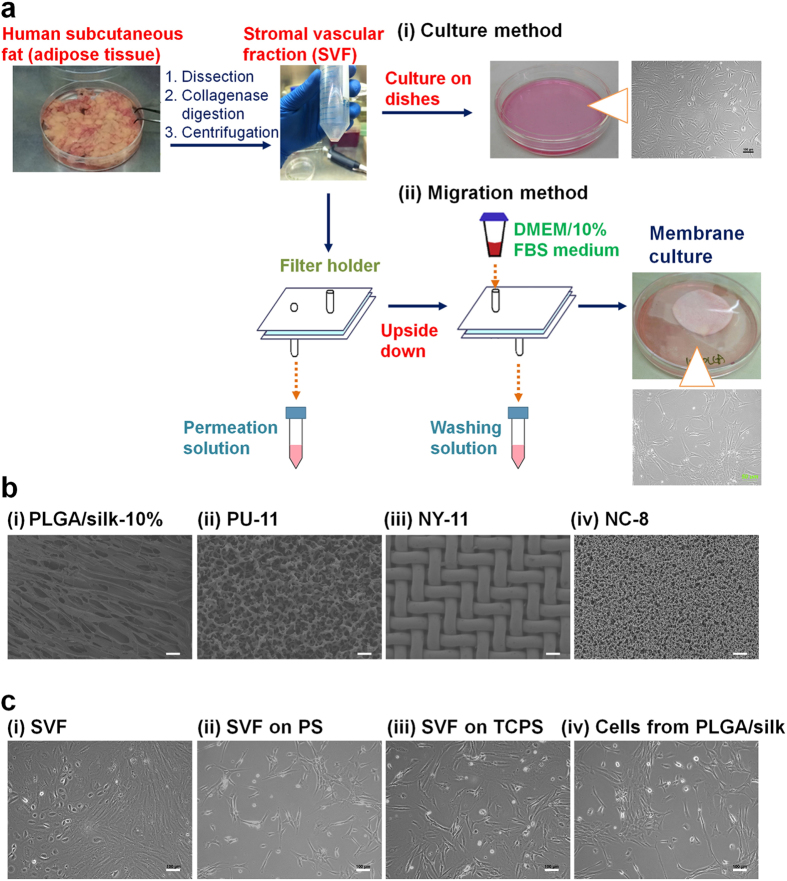
Purification of hADSCs from adipose tissue using the hybrid-membrane migration method. (**a**) Procedure of the hybrid-membrane migration method. The primary adipose tissue cell solution (SVF) was filtered through the membranes. Subsequently, a washing solution identical to the culture medium was passed through the membranes. The membranes were removed from the membrane holder and inserted into dishes containing culture medium. hADSCs migrated out from the membranes and were expanded in the dishes during culture. (**b**) Morphology of PLGA/silk-10% (i), polyurethane (PU-11) (ii), nylon mesh filter (NY-11) (iii) and nitrocellulose (NC-8) (iv) membranes used for the hybrid-membrane migration method. The scale bars indicate 100 μm (i) and 40 μm (ii-iv). (**c**) The morphology of the primary adipose tissue cells (SVF) (i), SVF cells cultured on untreated PS dishes (ii) and on TCPS dishes (iii) for 8 days, and the cells that migrated from PLGA/silk-10% membranes and were subsequently cultured for 15 days after SVF was permeated through the membranes (iv). The scale bars indicate 100 μm.

The isolation of hADSCs using the hybrid-membrane migration method was performed with the following procedures (Fig. 1a): (a) The primary adipose tissue cell solution (SVF), which was prepared by the collagenase digestion of human fat tissue, was filtered through the membranes. (b) Subsequently, a washing solution identical to the culture medium was passed through the membranes. (c) The membranes were removed from the membrane holder and inserted into dishes containing culture medium. hADSCs that migrated out from the membranes were cultured in culture medium.

Several membranes with a pore size larger than 8 μm were selected to perform the hybrid-membrane migration method. The membranes used in this study were PLGA/silk membranes with a pore size (*r*) = 18.2-24.4 μm and were originally designed from biodegradable materials to allow them to be used as scaffolds for transplantation into animals^16^. Commercially available synthetic membranes such as nitrocellulose (NC-8, *r* = 8 μm), nylon mesh filter (NY-11, *r* = 11 μm) and polyurethane (PU-11, *r* = 11 μm)^15^ membranes were also selected. The morphology of these membranes is shown in Fig. 1b.

hADSCs were purified using the conventional culture method and the hybrid-membrane migration method. The morphologies of the primary adipose tissue cells (SVF), SVF cultured on untreated polystyrene dishes for one passage (PS; hADSCs purified using the culture method), SVF cultured on tissue culture polystyrene dishes for one passage (TCPS; hADSCs purified using the culture method), and the cells that migrated out from PLGA/silk membranes-10% (PLGA/silk membranes prepared using 10 wt% of PLGA solution; hADSCs purified using the hybrid-membrane migration method) are shown in Fig. 1c. Several different cellular morphologies, e.g., spread cells, oval-shaped cells similar to a rugby ball and stretched cells, were observed in SVF. This indicates that the primary adipose tissue cells (SVF) are a heterogeneous cell population that contains a variety of cell types, including hADSCs. After 8 days of culture, the cellular morphology became more homogeneous, and most of the cells showed a spindle-shaped morphology (Fig. 1c (ii) and (iii), the culture method). The spindle-shaped cells were also observed after hADSCs purification by the hybrid-membrane migration method (Fig. 1c (iv)).

### Surface marker assay of hADSCs

**Figure 2:**
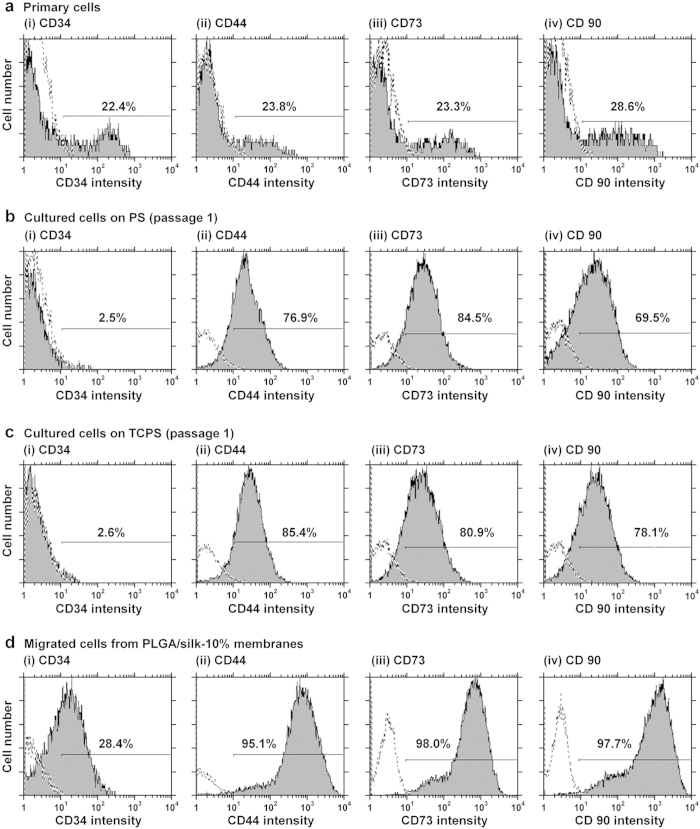
Purity of hADSCs isolated using the hybrid-membrane migration method. As analyzed by flow cytometry, the expression of CD34 (i) and MSC markers (CD44 [ii], CD73 [iii], and CD90 [iv]) on the primary adipose tissue cells (SVF) (**a**), first-passage SVF cells grown in untreated PS (**b**) and TCPS (**c**) dishes, and the cells that migrated out from PLGA/silk-10% membranes that were subsequently cultured for 15 days after SVF was permeated through the membranes (**d**). The dotted lines indicate cells labeled with the isotype controls.

An analysis of cell surface markers of the primary adipose tissue cell solution (SVF) and of the first passage of cells grown in TCPS and untreated PS dishes (conventional culture method used for the purification of hADSCs), as well as of the cells that migrated out from the membranes (hybrid-membrane migration method for the purification of hADSCs), was performed to evaluate the purity of the hADSCs in each cell solution. Figure 2 present representative flow cytometry results for the expression of CD34, CD44, CD73 and CD90 on (a) the primary adipose tissue cells (passage 0, SVF) (Fig. 2a), (b) the first-passage cells grown on PS (Fig. 2b), the first-passage cells grown on TCPS (Fig. 2c) and the cells that migrated from PLGA/silk-10% membranes (Fig. 2d). Fewer than 30% of the cells in the primary adipose tissue cell solution (SVF) expressed CD34, CD44, CD73 and CD90, which was similar to the results reported by previous studies^7^,^15^. In contrast, 70-85% of the cells that had been cultivated in PS and TCPS for one passage were positive for expression of CD44, CD73 and CD90, which was also similar to results reported by several researchers^7^,^10^,^11^,^15^. The expression of CD34 on the cells disappeared after culture on PS or TCPS dishes, which indicated that the conventional culture method cannot isolate hADSCs expressing CD34. An extremely high percentage of the cells that migrated from PLGA/silk-10% membranes (i.e., >95%) expressed MSC surface markers including CD44, CD73 and CD90. The percentage of migrated cells that also expressed CD34 was comparable to or higher than that of the primary adipose tissue cells (SVF).

Supplementary Fig. 1 shows representative flow cytometry results for the expression of CD34, CD44, CD73 and CD90 on the cells that migrated from PLGA/silk membranes prepared from different PLGA concentrations (i.e., PLGA/silk-3%, PLGA/silk-5%, PLGA/silk-10% and PLGA/silk-15%).

**Figure 3:**
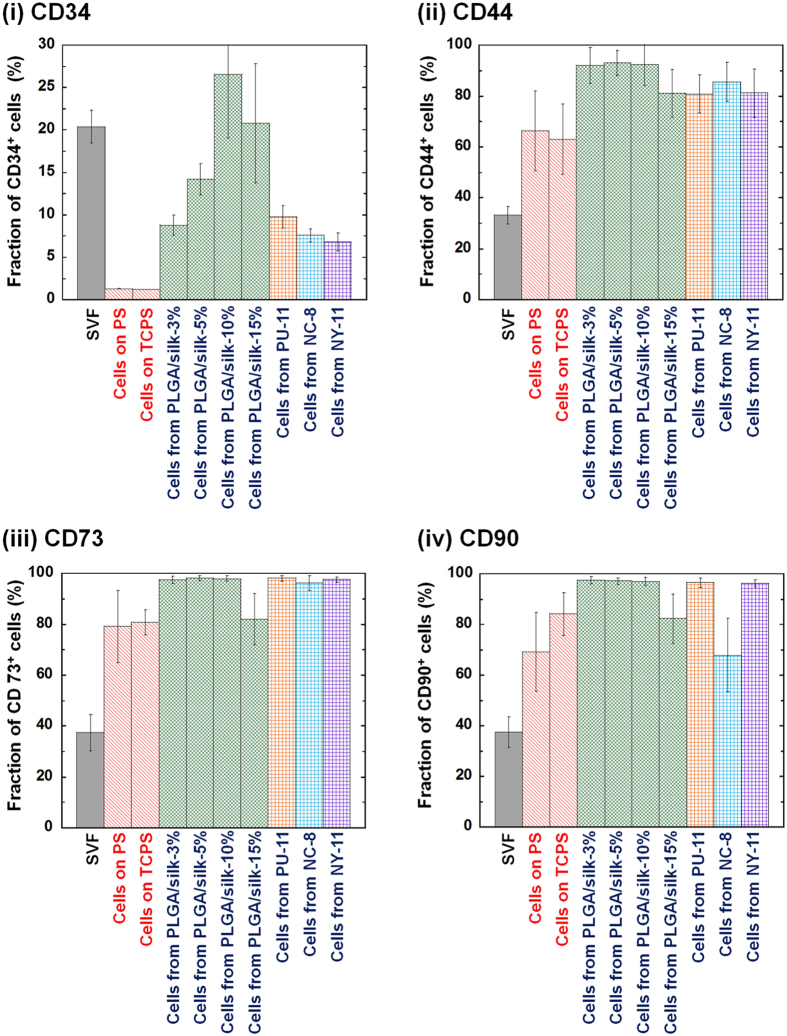
MSC marker expression of hADSCs isolated using the hybrid-membrane migration method. As analyzed by flow cytometry, the expression of CD34 (i), CD44 (ii), CD73 (iii) and CD90 (iv) on SVF cells, first-passage cells cultured on untreated PS and TCPS, and the cells that migrated out from PLGA/silk, PU-11, NC-8 and NY-11 membranes and were subsequently cultured on untreated PS dishes for 15 days after SVF was permeated through the membranes.

Figure 3 summarizes the expression of CD34, CD44, CD73 and CD90 on SVF, first-passage SVF cultured on PS and TCPS and the cells that migrated out from several types of membranes. It should be noted that 20-25% of the SVF cells expressed CD34, which categorized them not as hematopoietic stem cells but rather as endothelial progenitor cells^7^,^15^. The CD34 expression of the cells dramatically decreased after cultivation on both PS and TCPS for one passage, which is similar to previous results^7^. It is known to be difficult to isolate cells expressing CD34, which is co-expressed with mesenchymal stem cell markers, when the culture method is used^7^,^15^. However, hADSCs purified using the hybrid-membrane migration method did express CD34. More than 95% of the cells that migrated from PLGA/silk-3%, PLGA/silk-5% and PLGA/silk-10% membranes expressed CD73 and CD90, and more than 90% expressed CD44; these percentages of cells positive for MSC marker expression were higher than for the cells isolated using the conventional culture method.

It should be mentioned that not only do a high percentage of the cells that migrated from PLGA/silk express MSC surface markers, but each cell also expresses a high amount of MSC surface markers. The peak of CD44, CD73 and CD90 expression in the cells that migrated from PLGA/silk membranes (Fig. 2d and Supplementary Fig. 1) was much higher than that of SVF cells (Fig. 2a) and that of SVF cultured on both PS (Fig. 2b) and TCPS (Fig. 2c) for one passage. These results indicate that hADSCs purified using the hybrid-membrane migration method express much higher levels of MSC surface markers on each cell than hADSCs purified using the conventional culture method and the cells in SVF.

### Purification hADSCs using commercially available membranes

The ease of purifying hADSCs with high purity and high pluripotency would be increased if commercially available membranes, rather than hand-made PLGA/silk membranes, could be used for the hybrid-membrane migration method. Therefore, commercially available NC-8, NY-11 and PU-11 membranes were evaluated for the purification of hADSCs using the hybrid-membrane migration method.

Supplementary Fig. 2 shows representative flow cytometry results for the expression of CD44, CD73 and CD90 on the cells that migrated out from NC-8, NY-11 and PU-11 membranes. Figure 3 also summarizes the expression of CD34, CD44, CD73 and CD90 on the cells that migrated from NC-8, NY-11 and PU-11 membranes. More than 95% of the cells that migrated from NC-8, NY-11 and PU-11 membranes were positive for CD73 expression, which was higher than the percentage of cells purified using the conventional culture method (*p* < 0.05). More than 95% of cells that migrated out from NY-11 and PU-11 membranes were positive for CD90 expression, which was similar to number of positive cells that migrated out from PLGA/silk-3%, -5% and -10% membranes. From these results, the commercially available NY-11 membranes should be appropriate for use in the hybrid-membrane migration method because the cells that migrated from NY-11 were similar to those that migrated from PLGA/silk-3%, -5%, and -10% membranes. The hybrid-membrane migration method using NC-8, NY-11 and PU-11 membranes also successfully isolated hADSCs expressing CD34.

### Pluripotent gene assay of hADSCs purified by hybrid-membrane migration method

**Figure 4:**
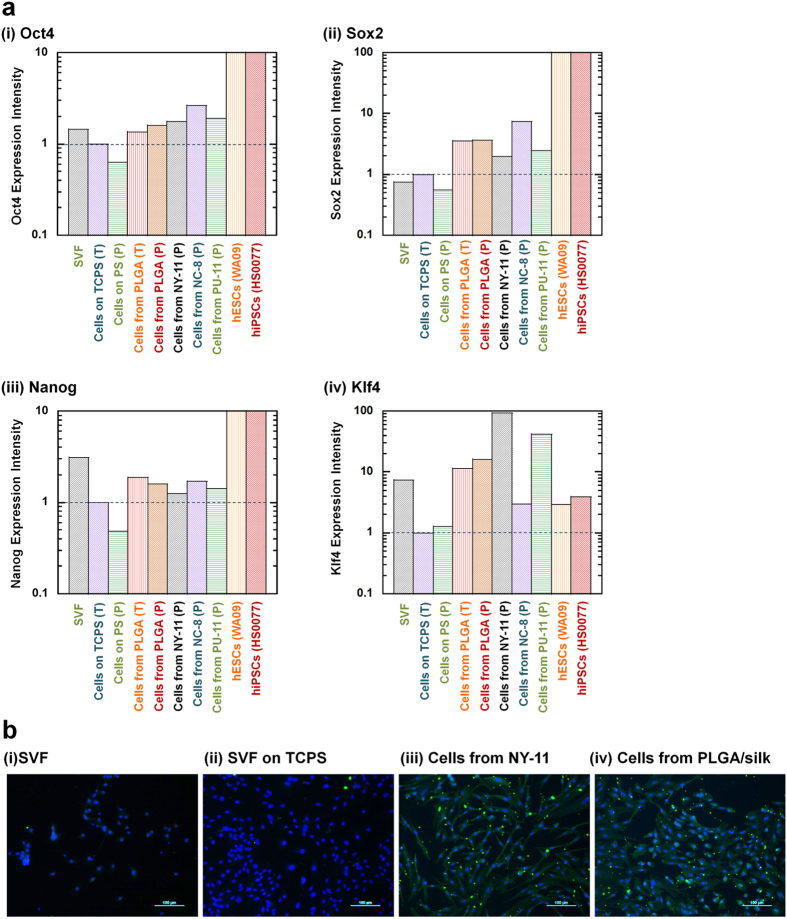
Pluripotency of hADSCs isolated using the hybrid-membrane migration method. (**a**) Relative gene expression levels of Oct4 (i), Sox2 (ii), Nanog (iii) and Klf4 (iv) as analyzed by qRT-PCR in the primary adipose tissue cells (SVF), the primary adipose tissue cells (SVF) cultured on untreated PS and TCPS dishes for 8 days (passage one), the cells that migrated out from PLGA/silk-10%, PU-11, NC-8 and NY-11 membranes and were subsequently cultured on untreated PS (P) and TCPS (T) dishes after SVF was permeated through the membranes, hESCs (WA09) and hiPSCs (HS0077). The gene expression was standardized to the expression in the primary adipose tissue cells (SVF) cultured on TCPS. (**b**) The expression of the pluripotency protein Oct4 in the primary adipose tissue cells (SVF) cultured on untreated PS dishes (i), the primary adipose tissue cells (SVF) cultured on TCPS dishes for 8 days (passage one) (ii), and the cells that migrated out from NY-11 (iii) and PLGA/silk-10% (iv) membranes and were subsequently cultured on untreated PS dishes after SVF was permeated through the membranes, as analyzed by immunostaining for Oct4. Oct4 expression is shown in green, and nuclear staining by Hoechst is shown in blue. The scale bars indicate 100 μm.

Based on the MSC surface marker results, we expected that hADSCs purified using the hybrid-membrane migration method might have a higher pluripotency than hADSCs purified using the conventional culture method. Therefore, the expression of the pluripotency genes *Oct4*, *Sox2*, *Nano*g and *Klf4* was evaluated by qRT-PCR in (a) the primary adipose tissue cell solution (SVF), (b) hADSCs purified using the hybrid-membrane migration method and (c) hADSCs purified using the conventional culture method with PS and TCPS dishes (as control experiments), as well as hESCs (WA09) and hiPSCs (HS0077) (as positive controls) (Fig. 4a). In hESCs and hiPSCs, the expression of *Oct4*, *Sox2* and *Nanog*, which are genes typically used to generate hiPSCs by transduction into somatic cells^2^,^4^, was found to be 2-3 orders of magnitude higher than that of hADSCs purified using the conventional culture method. Furthermore, the cells that migrated from any membrane (PLGA/silk, NY-11, NC-8 and PU-11) showed higher expression of *Oct4*, *Sox2*, and *Nanog* than hADSCs purified using the conventional culture method. It should be mentioned that *Klf4* expression in hADSCs purified using the membrane filtration method was higher than the expression in hESCs and hiPSCs. The elevation of *Klf4* gene expression in somatic cells is known to enhance the efficiency of the reprogramming of somatic cells into iPSCs^17^,^18^,^19^,^20^.

Immunostaining of the pluripotency marker Oct4 was also evaluated (Fig. 4b). hADSCs purified using the hybrid-membrane migration method were found to express higher levels of Oct4 protein than hADSCs purified using the conventional culture method. These results are consistent with the expression of pluripotency genes found in Fig. 4a.

In conclusion, the hybrid-membrane migration method is able to purify CD34-positive hADSCs from a primary fat tissue solution (SVF) with high purity and high pluripotency using a simple method without antibodies. Due to the high expression of the pluripotency genes *Oct4*, *Sox2*, *Klf4* and *Nanog*, hADSCs purified using the hybrid-membrane migration method should be a promising source of cells for hiPSC reprogramming that will require fewer transduction genes and/or can be reprogrammed with higher efficiency.

## Discussion

The membranes used in this study were originally developed for scaffolds composed of biodegradable PLGA and silk mesh where hADSCs were seeded for stem cell culture and immobilization. The scaffolds which did not coated with extracellular matrices (ECMs) such as collagen I were found not to be suitable for the scaffolds to culture and immobilize hADSCs in the scaffolds, but surprisingly, hADSCs were found to migrate out from the scaffolds. Synthetic polymeric scaffolds (membranes) without coating of ECMs seems not to be favor for hADSCs culture. Therefore, hADSCs having high mobility tends to escape from uncomfortable synthetic scaffolds to the outside of the synthetic scaffolds, which had high expression of MSC markers and higher pluripotent gene and protein expression compared to hADSCs purified by the conventional culture method as found in Fig. 2, 3, 4. The dead cells could not migrate out from the scaffolds and hADSCs being not so active remained inside of the scaffolds. This is the purification mechanism of hADSCs why the hybrid-membrane migration method can purify hADSCs from the primary adipose tissue solution (SVF) in this study.

We found that we could successively purify hADSCs by the hybrid-membrane filtration method using (a) PLGA/silk membranes with a pore size (*r*) = 18.2-24.4 μm, (b) nitrocellulose (NC-8, *r* = 8 μm), (c) nylon mesh filter (NY-11, *r* = 11 μm) and (d) polyurethane (PU-11, *r* = 11 μm). The porous membranes having variety of synthetic materials seem to be used in the hybrid-membrane migration method. However, porous membranes made of ECMs such as collagen scaffold or porous membranes coated with ECMs should be less effective to use in the purification of hADSCs by the hybrid-membrane migration method. This is because hADSCs tend to adhere on ECMs in the membranes and are hardly to migrate out from the membranes with less efficiency compared to hADSCs purified through the synthetic porous membranes. Another important characteristics of the membranes used in the hybrid-membrane migration method is the pore size of the membranes. Almost no permeation of the primary adipose tissue cell solution (SVF) was observed or high pressure was required for permeation of the primary adipose tissue cell solution, when the membranes having less than 8 μm were used in the hybrid-membrane migration method (e.g., nitrocellulose membranes having *r* = 5 μm). When the nylon mesh filter having *r* = 20 μm was used, we could collect the membrane filtration method and the yield of hADSCs became less than half compared to that purified using NY-11. Furthermore, the yield of hADSCs became less than 10% using the nylon mesh filter having having *r* = 40 μm compared to that purified using NY-11 primary adipose tissue cell solution. Therefore, the optimal pore size of the membranes used in the hybrid-membrane filtration method seems to be in the range of 8-25 μm.

## Methods

**Preparation of the adipose tissue cell solution.** The experiments in this study were approved by the ethics committees of the National Central University, the Taiwan Landseed Hospital (IRB-13-05), and the Cathay Medical Research Institute (CT099012). All experiments were performed in accordance with all applicable and relevant institutional and governmental regulations and guidelines during this study. Adipose tissue was isolated from the fat pads of human patients from the omentum and near the intestines (50-87 years old, ten people) with informed consent. The adipose tissue cell solution (SVF, stromal vascular fraction) was generated using a previously reported method^9^,^15^,^21^. The fat tissue was dissected and washed with phosphate-buffered saline (PBS) to remove blood and impurities. The adipose tissue was then minced into small pieces (approximately 2 mm^3^) and digested with 2.5 mg/mL type I collagenase (C0130, Sigma-Aldrich, St. Louis, MO) at 37 °C for 60 min. The enzymatic activity was neutralized with Dulbecco’s modified Eagle’s medium (DMEM, D5523, Sigma-Aldrich, St. Louis, MO) containing 10% fetal bovine serum (FBS; 04-001-1, Lot 551035, Biological Industries Ltd., Israel). The digested solution was centrifuged at 1200 × g for 7 min. The resulting cells were suspended for 2 min in erythrocyte lysis buffer (pH 7.4, 154 mM NH_4_Cl, 20 mM Tris) to remove red blood cells, and the solution was subsequently neutralized with DMEM culture medium containing 10% FBS. The cell solution was centrifuged at 1200 × g for 7 min. The resulting cells were resuspended in DMEM culture medium containing 10% FBS to yield the primary adipose tissue cell solution (SVF)^9^,^21^.

The number of hADSCs in the primary adipose tissue cell solution was determined using flow cytometry with antibodies to CD34 (FITC mouse anti-human CD34, IM1870U, Beckman Coulter, Inc., Marseille, France), CD44 (FITC mouse anti-human CD44, IM1219U, Beckman Coulter, Inc., Marseille, France), CD73 (PE mouse anti-human CD73, 550257, BD Biosciences, San Jose, CA), CD90 (PE mouse anti-human CD90, IM1840U, Beckman Coulter, Inc., Marseille, France), and appropriate isotype controls (PE mouse anti-human IgG1, 733179, and FITC mouse anti-human IgG1, 41116015, Beckman Coulter, Inc., Marseille, France). The total cell number in the primary adipose tissue cell solution was also determined by flow cytometry (Coulter EPICS™ XL, Beckman Coulter, Inc., Marseille, France) after staining with 7-AAD (A07704, Beckman Coulter, Inc., Marseille, France). The primary adipose tissue cell solution was purified using the conventional culture method^8^,^15^,^22^ and the hybrid-membrane migration method developed in this study.

**Purification of hADSCs using the culture method.** hADSCs were purified using the conventional culture method to compare the purity of hADSCs with hADSCs purified by the hybrid-membrane migration method. The cells in the primary adipose tissue cell solution (SVF) were plated in tissue culture polystyrene dishes (TCPS, 430167, Corning Incorporated, USA) or untreated polystyrene dishes (PS, 351029, Falcon^R^, BD Biosciences, USA) at approximately 10,000 cells/cm^2^ in DMEM containing 10% FBS at 37 °C in a 5% CO_2_ incubator. When the cells reached approximately 80-90% confluence (8-10 days), the cells were trypsinized with a 0.25% trypsin-EDTA solution (25200-056, Invitrogen Corporation, Carlsbad, CA), followed by centrifugation at 1500 rpm for 5 min. The hADSCs thus obtained were resuspended in DMEM containing 10% FBS and were used for analysis (first-passage hADSCs)^15^,^21^. The number of hADSCs in the cell culture medium at the first passage was determined by flow cytometry using antibodies against CD34, CD44, CD73 and CD90 and the appropriate isotype controls. The total cell number in the cell culture medium at the first passage was also determined by flow cytometry^9^,^21^.

### Preparation of PLGA/silk screen hybrid membranes

PLGA (poly[lactide-co-glycolic acid])/silk screen hybrid membranes were prepared using the freeze-extraction method (Supplementary Fig. 3)^9^,^16^,^21^,^23^. PLGA (lactide:glycolic=75:25, P1941, Sigma-Aldrich, St. Louis, MO, USA) was dissolved in N,N-dimethyl sulfoxide (DMSO) to produce a 3, 5, 10 or 15 wt% PLGA solution. The PLGA solution was heated at 80 °C for 20 min until homogeneous and subsequently cooled to room temperature. Next, the silk screen (170 mesh size) with square dimensions of 3.3 cm × 3.3 cm was inserted into a glass petri dish (50 mm diameter) that was coated with high-vacuum grease (1658832, Dow Corning Corporation, Midland, MI, USA) to prevent membrane adherence^9^,^21^. Subsequently, 4.5 mL of the PLGA solution was injected into the glass dishes containing the silk screen and frozen at −20 °C for 24 h. The solvent contained in the frozen PLGA solution with the silk screen was removed by immersing the frozen PLGA solution into a 75% ethanol solution at −20 °C for three days and exchanging with 75% ethanol twice a day. The DMSO solvent was extracted and replaced with a 75% ethanol solution during this process, and the PLGA/silk screen hybrid membranes were generated^21^. The PLGA/silk screen hybrid membranes were subsequently allowed to dry in a fume hood for three days and then dried under vacuum for two days to remove the aqueous ethanol solution. The PLGA/silk screen hybrid membranes thus prepared were sterilized by UV light for one day before being used in the following hybrid-membrane migration method^9^.

Purification of hADSCs by the hybrid-membrane migration method. hADSCs were purified from the primary adipose tissue cell (SVF) solution by migration from membranes. The membranes used in this study were: (a) PLGA/silk screen hybrid membranes prepared from 3, 5, 10, or 15 wt% PLGA solution, which are denoted as PLGA/silk-3%, PLGA/silk-5%, PLGA/silk-10%, or PLGA/silk-15%, respectively), (b) polyurethane-forming membranes with a pore size of 11 μm^15^, (c) nitrocellulose membranes with a pore size of 8 μm (NC-8, SCWP, EMD Millipore, Merck KGaA, Darmstadt, Germany), and (d) nylon mesh filter membranes with a pore size of 11 μm (NY-11, EMD Millipore, Merck KGaA, Darmstadt, Germany). Some physical characteristics of the membranes are summarized in Supplementary Table 1. A membrane holder and permeation method similar to those reported in previous work^9^,^15^,^21^ were used in this study.

In the hybrid-membrane migration method, 18 mL of the primary adipose tissue cell solution (SVF) with a total cell number of 1.0 × 10^6^ was passed through the membranes at 25 °C with a filtration rate of 1 mL/min after the membranes were loaded into the membrane filtration holder, as described in Fig. 1a. The numbers of hADSCs in the permeation solution and the feed solution were determined using flow cytometry with antibodies against CD34, CD40, CD73 and CD90 and the appropriate isotype controls.

Following cell filtration, the membranes were placed upside down inside the membrane holder, and the washing solution that consisted of DMEM containing 10% FBS was permeated through the membranes using the same apparatus at a filtration speed of 1 mL/min at 25 °C (Fig. 1a). This step was performed to wash the membranes and remove the cells that were weakly attached to the membranes into the washing solution. The number of hADSCs in the washing solution was determined using flow cytometry with antibodies against CD34, CD40, CD73 and CD90 and the appropriate isotype controls.

After permeation of the washing solution, the membranes were subsequently removed from the membrane holder and inserted into culture medium (DMEM containing 10% FBS) on TCPS or PS dishes. The residual cells on the membranes started to migrate out from the membranes into the culture dishes (TCPS or PS) and were incubated for 8-15 days at 37 °C in a 5% CO_2_ incubator until the cells became confluent on the culture dishes. When the cells (hADSCs) reached approximately 80-90% confluence, the cells were trypsinized with a 0.25% trypsin-EDTA solution (25200-056, Invitrogen Corporation, Carlsbad, CA), followed by centrifugation at 1500 rpm for 5 min. The hADSCs thus obtained were resuspended in DMEM containing 10% FBS and were used for analysis. The number of hADSCs in the cell culture medium was determined by flow cytometry using antibodies against CD34, CD44, CD73, and CD90 and appropriate isotype controls. The total cell number in the cell culture medium was also determined by flow cytometry.

**hESC and hiPSC culture.** The hESC line WA09 (H9) was obtained from the WiCell Research Institute (Madison, WI). The hiPSC line, HPS0077, was acquired from Riken Cell Bank (Tsukuba, Japan). Matrigel (BD Biosciences, San Jose, CA) was diluted 1:1 in DMEM/F12 medium (11330-057, Invitrogen Corporation, Carlsbad, CA) and used to coat polystyrene tissue culture dishes for one hour. hESCs and hiPSCs were seeded and cultured on Matrigel-coated dishes in the chemically defined medium mTeSR1 (Stem Cell Technologies, Vancouver, Canada) at 37 °C and 5% CO_2_ with daily medium exchange^24^.

### Pluripotency assay of hADSCs

The expression levels of the pluripotency genes *Oct4*, *Sox2*, *klf4*, and *Nanog* were analyzed by qRT-PCR using conventional methods^25^,^26^,^27^. Briefly, after the cells were harvested, RNA was extracted using the TRI-Reagent kit (Sigma-Aldrich, St. Louis, MO, USA) according to the manufacturer’s instructions. The isolated RNA was treated with DNase (Ambion; Austin, TX) to remove any traces of contaminating DNA^21^. RNA (2 μg) was reverse-transcribed to produce cDNA using reverse transcriptase (SuperScript III First-Strand synthesis system for RT-PCR; Invitrogen, Carlsbad, CA). This cDNA was then used as a template for polymerase chain reaction (PCR) amplification. Probes of *Oct4* (Hs01895061_u1), *Sox2* (Hs00602736_s1), *Nanog* (Hs02387400_g1), *Klf4* (Hs00382393_CE) and *GAPDH* (glyceraldehyde-3-phosphate dehydrogenase, Hs03929097_g1) were purchased from Life Technologies (Carlsbad, California). PCR amplification was performed using Taq DNA polymerase (TITANIUM^TM^ Taq PCR Kit, Clontech Laboratories, Inc., Madison, WI) and a thermocycler (TPersonal, Biometra, GmbH, Goettingen, Germany). Each sample (*n* = 3) was tested in duplicate, and the expression level of the GAPDH housekeeping gene was used as a control to normalize the results^28^.

Immunostaining of Oct4 on the cells was performed following the conventional protocol^29^,^30^. Cells on dishes were fixed with paraformaldehyde and then incubated with mouse anti-human Oct4 antibody (1:100, ab27985, Abcam, Cambridge, MA). Subsequently, the cells were washed with PBS and incubated with the secondary antibody Alexa Fluor 488-anti-mouse IgG (1:50, A21202, Invitrogen, Grand Island, NY). The cells were also incubated with Hoechst 33342 (1:50, PA-3014, Lonza Inc., Allendale, NJ). The stained cells were analyzed using fluorescence microscopy with the appropriate filters (Eclipse Ti-U fluorescence inverted microscope, Nikon Instruments, Inc., Tokyo, Japan).

### Statistical analysis

All of the quantitative results were obtained from four samples. The data are expressed as the mean ± SD. Statistical analyses were performed using the unpaired Student’s *t*-test in Excel (Microsoft Corporation). Probability values (*p*) less than 0.05 were considered statistically significant.

## Author Contributions

A. H. organized the experimental project, designed the hybrid-membrane migration method and wrote the manuscript. C.-T. W. also designed the membrane migration method and performed hADSC purification experiments. S. S. K. and Y. C. analyzed hADSCs by flow cytometry. M. A. M. and A. A. A. performed qRT-PCR experiments. Q.-D. L., H. H. L. and S. T. H. analyzed the data. G. J. W. and A. U. discussed the data and manuscript.

## Additional Information

**How to cite this article**: Higuchi, A. *et al.* A hybrid-membrane migration method to isolate high-purity adipose-derived stem cells from fat tissues. *Sci. Rep.* **5**, 10217; doi: 10.1038/srep10217 (2015).

## Supplementary Material

Supplementary Information
