# Supplementary material for: A hybrid-membrane migration method to isolate high-purity adipose-derived stem cells from fat tissues
Source: Sci Rep. 2015 May 13;5:10217. doi: 10.1038/srep10217 (PMC4429558; doi:10.1038/srep10217)
Supplement: Supplementary Information [file srep10217-s1.pdf]

Supplementary Material for - A hybrid-membrane migration method to isolate high-purity adipose-derived stem cells from fat tissues

Akon Higuchi[1,2,3,4], Ching-Tang Wang[1], Qing-Dong Ling[5,6], Henry Hsin-chung Lee[7], S. Suresh Kumar[8], Yung Chang[9], Abdullah A. Alarfaj[2], Murugan A. Munusamy[2], Shih-Tien Hsu[10], Gwo-Jang Wu[11], Akihiko Umezawa[3]

[1]Department of Chemical and Materials Engineering, National Central University, Jhong-li, Taoyuan, Taiwan

[2]Department of Botany and Microbiology, King Saud University, Riyadh, Saudi Arabia

[3]Department of Reproduction, National Research Institute for Child Health and Development, Tokyo, Japan

[4]Nano Medical Engineering Laboratory, RIKEN, Wako, Saitama, Japan

[5]Institute of Systems Biology and Bioinformatics, National Central University, Jhong-li, Taoyuan, Taiwan

[6]Cathay Medical Research Institute, Cathay General Hospital, Taipei, Taiwan

[7]Department of Surgery, Cathay General Hospital, Taipei, Taiwan

[8]Department of Medical Microbiology and Parasitology, Universities Putra Malaysia, Slangor, Malaysia

[9]Department of Chemical Engineering, R&D Center for Membrane Technology, Chung Yuan Christian University, Jhong-li, Taoyuan, Taiwan

[10]Department of Internal Medicine, Taiwan Landseed Hospital, Pingjen, Taoyuan, Taiwan

[11]Graduate Institute of Medical Sciences and Department of Obstetrics & Gynecology, Tri-Service General Hospital, National Defense Medical Center, Taipei, Taiwan

SUPPLEMENTARY FIGURES

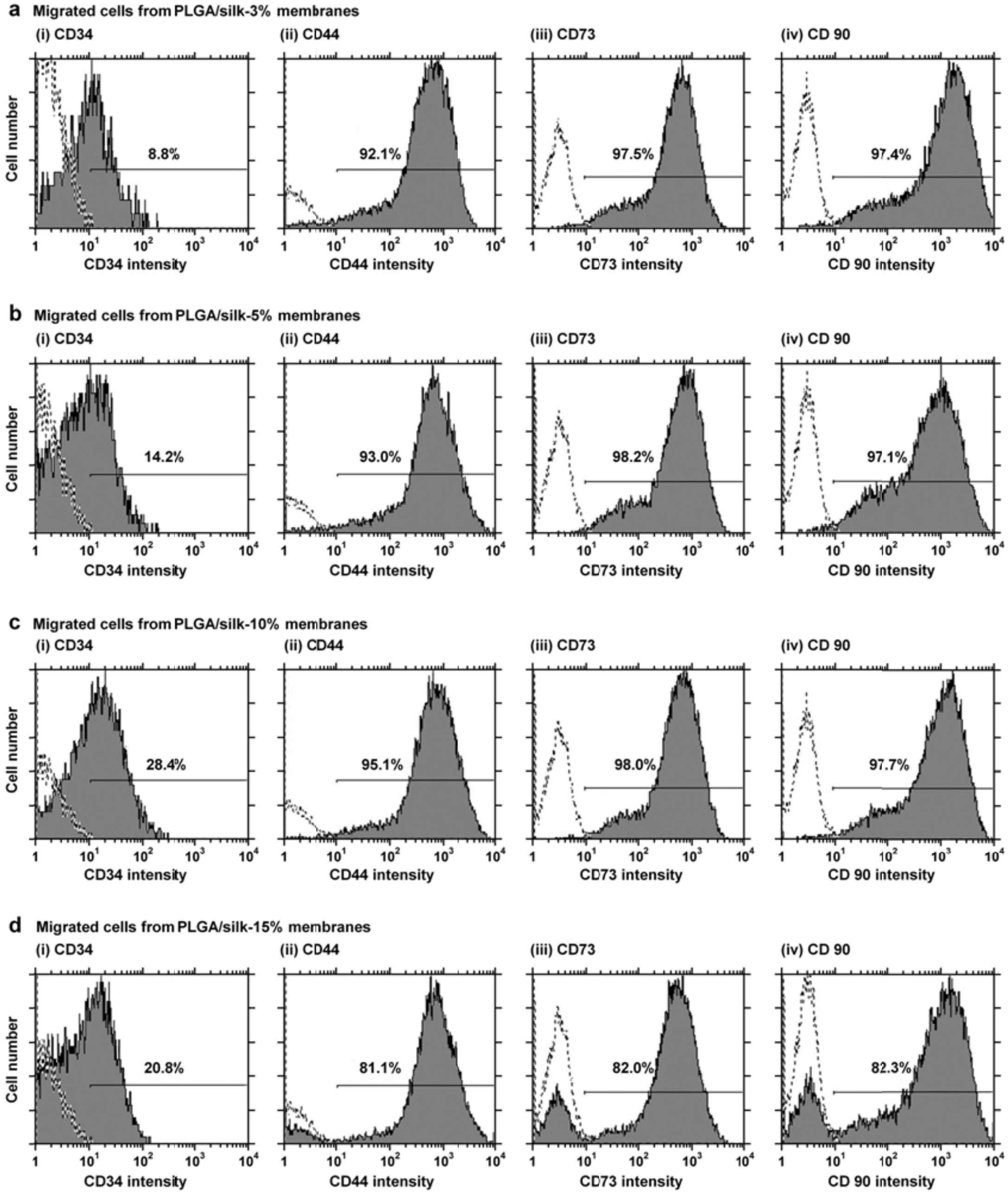

Supplementary Figure 1. CD34 (i) and MSC markers (CD44 (ii), CD73 (iii), and CD90 (iv)) expression analyzed by flow cytometry. Flow cytometry scattergrams of the migrated cells from (**a**) PLGA/silk-3%, (**b**) PLGA/silk-5%, (**c**) PLGA/silk-10% and (**d**) PLGA/silk-15% membranes and subsequently cultured for 15 days after SVF was permeated through the membranes. The dotted lines indicate cells labeled with the isotype controls.

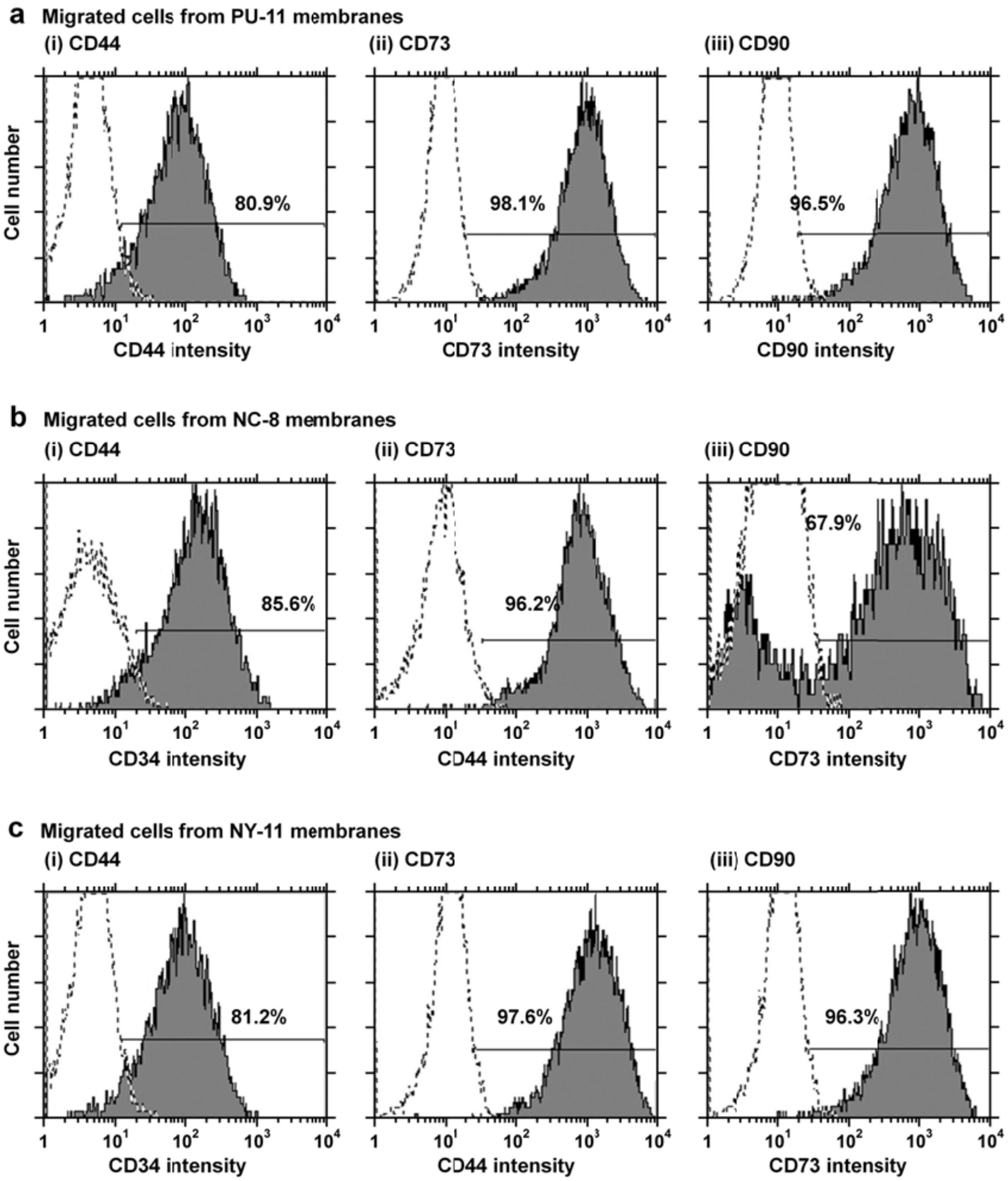

Supplementary Figure 2. MSC markers (CD44 [i], CD73 [ii], and CD90 [iii]) expression analyzed by flow cytometry. Flow cytometry scattergrams of the migrated cells from (**a**) PU-11, (**b**) NC-8 and (**c**) NY-11membranes and subsequently cultured for 15 days after SVF was permeated through the membranes. The dotted lines indicate cells labeled with the isotype controls.

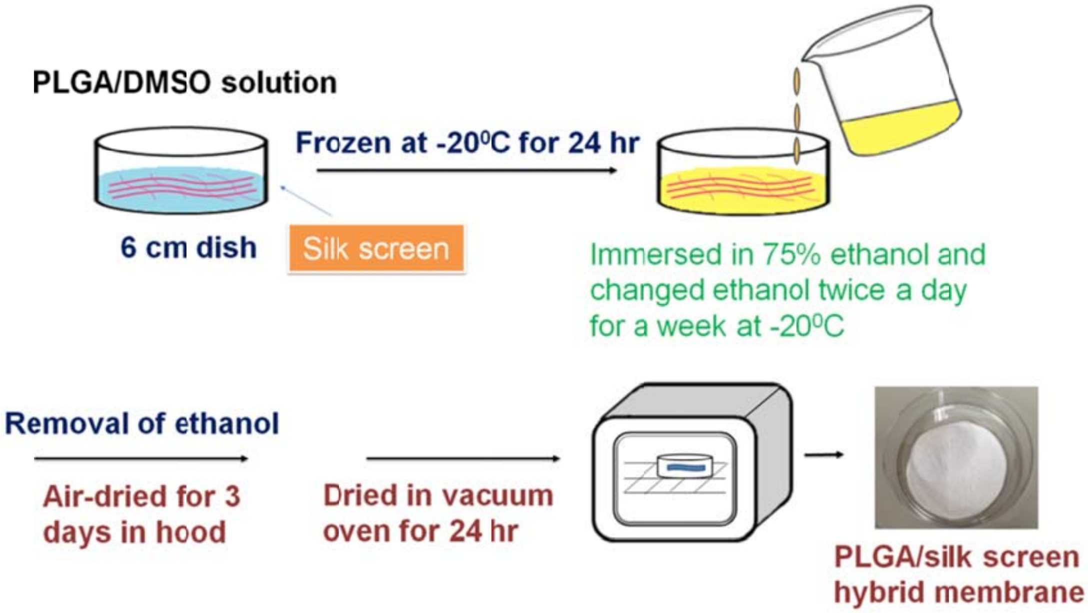

Supplementary Figure 3. Manufacturing procedures of the PLGA/silk membranes prepared by the freeze-extraction method.

SUPPLEMENTARY TABLE

Supplementary Table 1 Characteristics of membranes used in this study.

Membranes	Materials	Pore size (μm)	Porosity (%)	Thickness (μm)
Silk-PLGA-3%	Silk & PLGA	24.4		
Silk-PLGA-5%	Silk & PLGA	22.4		
Silk-PLGA-10%	Silk & PLGA	20.8		
Silk-PLGA-15%	Silk screen & PLGA	18.2		
PU-11	Polyurethane	11.0	86	1200
NC-8	Nitrocellulose	8.0	84	135
NY11	Nylon	11.0	6	65
